# One-step removal of nasobiliary tube and pancreatic stent with a new linked-stent approach: a green tip to avoid additional procedure

**DOI:** 10.1055/a-2748-1270

**Published:** 2025-12-17

**Authors:** Rui Tao, Cong Chen, Jinyan Liu, Xia Ou, Leida Zhang, Danqing Liu

**Affiliations:** 1 Weight Management Center, Bishan Hospital, Chongqing University of Chinese Medicine, Chonqqing, China; 2 643861 Department of Hepatobiliary Surgery, Bishan Hospital of Chongqing Medical University, Bishan Hospital of Chongqing, Chongqing, China; 3 388288 Department of Hepatobiliary Surgery, Southwest Hospital, Third Military Medical University (Army Medical University), Chongqing, China

Pancreatic duct stent placement is one of the effective methods for preventing post-endoscopic retrograde cholangiopancreatography (ERCP) pancreatitis 1 . However, an additional endoscopic procedure is needed to remove the pancreatic duct stent, which increases the pain and economic burden for patients. In China, endoscopic nasobiliary drainage is the standard approach for biliary drainage following the endoscopic procedure. Herein, we developed a technique to attach the pancreatic duct stent to a nasobiliary tube, thereby enabling their removal in a single procedure ( Video 1 ).

A novel technique that connects the nasobiliary drain and the pancreatic duct stent to facilitate their simultaneous removal.Video 1

A 45-year-old female patient with common bile duct stones underwent ERCP for stone extraction. During the endoscopic procedure, the guidewire repeatedly accessed the pancreatic duct unintentionally. Consequently, the double-guidewire technique was employed, which ultimately achieved successful selective biliary cannulation. Following the complete clearance of the stones, nasobiliary drain and pancreatic stent placement were performed. Two separate sutures were secured at the distal end of the pancreatic stent and the tip of the nasobiliary catheter, respectively ( Fig. 1 **a, b** ). The two sutures were then connected ( Fig. 1 **c** ). Following pancreatic duct stent placement over the guidewire, both guidewires were simultaneously inserted into an 8.5-Fr nasobiliary catheter ( Fig. 1 **d** ). After placing the pancreatic duct stent using a nasobiliary tube ( Fig. 2 **a, b** ), the pancreatic duct guidewire was withdrawn, and the nasobiliary tube was positioned over the bile duct guidewire ( Fig. 2 **c, d** ). The nasobiliary drain and pancreatic duct stent were removed simultaneously 48 hours postoperatively ( Fig. 3 ).

**Fig. 1:**
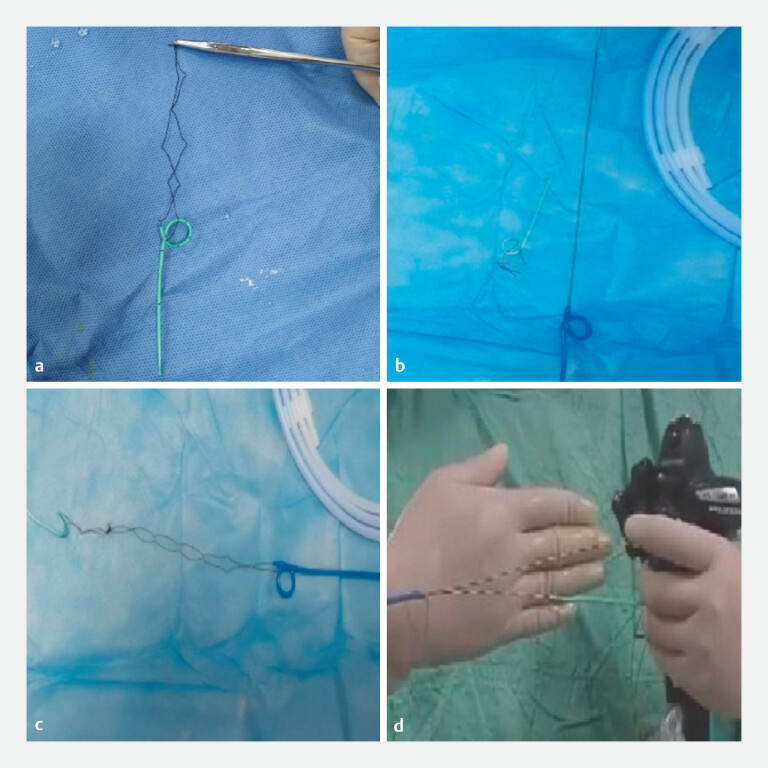
A novel connection technique between the nasobiliary catheter and the pancreatic stent. **a** Silk sutures of 10 cm were secured to the distal end of the pancreatic stent. **b** Silk sutures of 20 cm were secured to the tip of the nasobiliary catheter. **c** The pancreatic stent and the nasobiliary catheter were linked together via two sutures. **d** Following pancreatic duct stent placement over the guidewire, both guidewires were simultaneously inserted into an 8.5-Fr nasobiliary catheter.

**Fig. 2:**
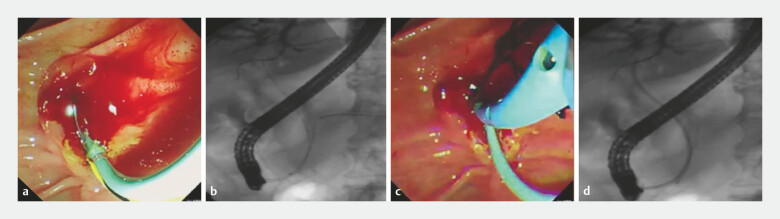
Placement of the pancreatic duct stent and nasobiliary drain. **a, b** The pancreatic duct stent was placed by the nasobiliary tube. **c, d** Nasobiliary drainage was performed lastly.

**Fig. 3:**
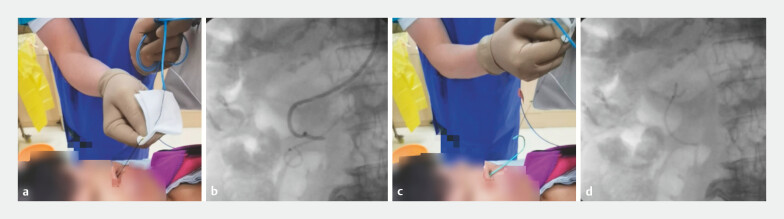
The nasobiliary cather and the pancreatic duct stent were successfully removed simultaneously.

This innovative nasobiliary-pancreatic stent connection technique allows for the safe and efficient removal of both devices at once, eliminating the need for an addition procedure. This approach not only decreases the exposure of both doctors and patients to radiation but also reduces the consumption of medical supplies. Notably, the suture connecting the nasobiliary tube and pancreatic duct stent should be of adequate length to facilitate their simultaneous and successful deployment. In this study, the length of the suture is based on the length of the extra-hepatic bile duct.

Endoscopy_UCTN_Code_TTT_1AR_2AZ

## Notes

Endoscopy E-Videos https://eref.thieme.de/e-videos
*E-Videos* is an open access online section of the journal *Endoscopy* , reporting on interesting cases and new techniques in gastroenterological endoscopy. All papers include a high-quality video and are published with a Creative Commons CC-BY license. Endoscopy E-Videos qualify for HINARI discounts and waivers and eligibility is automatically checked during the submission process. We grant 100% waivers to articles whose corresponding authors are based in Group A countries and 50% waivers to those who are based in Group B countries as classified by Research4Life (see: https://www.research4life.org/access/eligibility/ ). This section has its own submission website at https://mc.manuscriptcentral.com/e-videos .
